# Phenotypic characterization and analysis of genetic diversity between commercial crossbred and indigenous chickens from three different agro-ecological zones using DArT-Seq technology

**DOI:** 10.1371/journal.pone.0297643

**Published:** 2024-05-02

**Authors:** Kwaku Adomako, Selorm Sovi, Bismark Kyei, Jacob Alhassan Hamidu, Oscar Simon Olympio, Samuel E. Aggrey

**Affiliations:** 1 Department of Animal Science, Kwame Nkrumah University of Science and Technology, Kumasi, Ghana; 2 Nutrigenomics Laboratory, Department of Poultry Science, University of Georgia, Athens, Georgia, United States of America; 3 Farm Animal Genetic Resources Exploration and Innovation Key Laboratory of Sichuan Province, College of Animal Science and Technology, Sichuan Agricultural University, Chengdu, China; Ain Shams University Faculty of Agriculture, EGYPT

## Abstract

Indigenous and were used to study genetic diversity and population structure analyses. Polymorphism information content (PIC) values ranged from 0.0 to 0.5, with 21,285 SNP markers (35%) being in the lowest PIC value range (0 to 0.15) while 13,511 (commercial chickens have developed unique adaptations to their environments, which may include nutrition, pathogens, and thermal stress. Besides, environmental pressures and artificial selection have generated significant genome-wide divergence in chickens, as those selection pressures contribute a considerable evolutionary force to phenotypic and genotypic differentiation. Herein, we determined genomic diversity of indigenous chickens from semi-deciduous rainforest (SDR), coastal savannah (CS) and Guinea savannah (GS) agro-ecological zones (AEZs) in Ghana and commercial crossbreds (CC) reared at the Kwame Nkrumah University of Science and Technology (KNUST). We generated SNP markers from 82 chickens (62 indigenous chicken ecotypes and 26 commercial crossbred ecotype) using DArT-Seq technology. A total of 85,396 SNP markers were generated and after filtering the data, 58,353 markers 21%) were in the highest PIC value range (0.45 to 0.50). The CC were more genetically diverse than the indigenous birds, with the highest expected heterozygosity value of 0.220. Between the commercial crossbreds population and the indigenous ecotypes, pairwise F_ST_ values were estimated to be 0.105 between CS, 0.096 between SDF, and 0.133 between GS. Furthermore, PCA analysis showed that the CC, SDF and GS chickens clustered together and are genetically distant from the commercial crossbred. We herein show that chickens from the AEZs studied can be considered as one population. However, due the abundance of agro-byproducts in the SDR compared to the CS and GS, chickens from the SDR AEZ had better growth compared to their counterparts. It is suggested that the genetic diversity within the local ecotypes could form the basis for genetic improvement.

## Introduction

Chicken (*Gallus gallus*) is one of the most common domesticated species, as it plays a key role in agricultural and biomedical research fields. In Ghana, small-scale and commercial chicken production contribute significantly to human livelihood and food security of poor households [1, 2]. In addition, their impact goes beyond the provision of food, cash income, and employment, as they also serve as a means of capital accumulation and are valued in the religious and sociocultural lives of people [3, 4]. Chickens are found on all habitable continents, agro-ecological zones (AEZs), and their morphology and traits vary, which may affect their genetic makeup [5, 6]. Indigenous chicken ecotypes in Ghana are reared in the backyard and rely primarily on scavenging for their nutrition. Household scraps, crop residues and other agro-byproducts are their main sources of nutrition. Green vegetation, insects and worms also form part of their diet through scavenging.

Chicken phenotypic traits play a crucial role in various aspect of chicken production and management. Phenotypic traits refer to the observable characteristics of an organism. These traits have significant importance for farmers, breeders, and consumers alike [7, 8]. According to some studies, a challenging environment can shape the genomic landscape that underlies a population’s adaptation to climate and resources [9, 10]. Li et al. [11] stated that environmental pressures and artificial selection can generate significant genome-wide divergence in chickens, as those selection pressures contribute a considerable evolutionary force to phenotypic and genotypic differentiation. Phenotypic variation is controlled by genetic variation, environmental variation and interaction between heredity and environment. Therefore, improvement in husbandry practices and genetic potential of indigenous chickens could be a sustainable intervention to increase the productivity of indigenous chickens and enhance their conservation [12, 13]. Understanding the genetic diversity and differentiation of chickens commonly found in Ghana would provide the basis for genetic conservation and/or genetic improvement. In the last two decades, Diversity Arrays Technology (DArT) has produced a high-throughput marker technique called DArTseq based on genotyping-by-sequencing (GBS) to sequence the most informative representations of genomic DNA samples [14, 15]. DArTseq^™^ produces a fairly simple and accurate source of genetic data, which can also be used further for additional population analysis [16]. Furthermore, it integrates the genome complexity reduction method [17] with next-generation sequencing (NGS) approach. The technology merges high-throughput processing of DNA to produce complexity-reduced genome representations using combinations of restriction enzymes to select the optimal fraction of the genome for sequencing with rigorous analytical procedures [16]. This application has been successfully established in several plant [18, 19] and animal species [20, 21]. In addition, DArTseq has been applied to a wide range of species and applications, including the study of inter- and intra-specific genetic diversity and relationships, genetic mapping, genome-wide association studies, and genomic selection [22–25]. DArTseq produces single nucleotide polymorphism (SNP) markers that have been successfully applied for genetic structure analysis in several animal species [21, 26, 27].

A cascade of genes drives the traits of economic importance across the chicken genome [5]. Consequently, breeding programs and conservation techniques require genomic profiling of varied genetic animal resources and the identification of potential parents for future generations. The use of whole genome genetic markers to explore genetic diversity and uncover the genetic basis for specific traits in many chicken breeds has been studied quite extensively [26, 41, 49]. Assessment of genetic diversity based on whole genome genetic markers generated from high-throughput sequencing data has high accuracy than those based on platforms with few markers. However, the previous genomic study on the indigenous chicken population in Ghana was limited, and employed sequencing platforms with few markers such as simple sequence repeat markers (SSR markers) [28]. This study is envisaged to apply DArT markers to the genomic sequencing of chickens; and this is a novel approach not only in Ghana but globally.

The present study aimed to employ DArTseq SNPs to identify the genomic diversity of chickens within three AEZs and commercial crossbreds; and also characterize the phenotypic diversity between chickens from the three different AEZs and commercial crossbred chickens. A comprehensive and deep understanding of the genome underpinnings of the indigenous and commercial crossbreds could reveal the genetic diversity and population structure of these chickens in different AEZs. They can serve as the basis for genetic conservation and/or genetic improvement.

## Materials and methods

### Ethics statement

All the procedures for this experiment were conducted under a protocol approved by the Institutional Ethical Committee at Kwame Nkrumah University of Science and Technology (KNUST), Ghana with Certificate number KNUST-0004.

### Sampling of the indigenous and commercial crossbred

We randomly selected 104 birds for this experiment. The sampled birds were comprised of the indigenous free roaming chicken ecotypes and commercial crossbreds kept at the Kwame Nkrumah University of Science and Technology (KNUST) research station. As shown in S1 Table, a total of seventy-two (72) indigenous chickens were randomly selected, comprising 36 males and 36 females. We sampled 12 males and 12 females from each AEZ (Guinea Savannah, Semi-Deciduous Rainforest, and the Coastal Savannah agro-ecological). In addition, 16 male and 16 female commercial crossbreds were sampled from a crossbred population housed at KNUST (S2 Table) [29]. The crossbreds constitute an exotic Lohmann breed mated randomly with indigenous ecotypes. Since then, the various first filial males have been backcrossed to the Lohmann female for six successive generations. Hereafter, the Lohman x indigenous ecotype would be referred to as Commercial Crossbreds (CC).

### Description of the ecological zones

**Fig 1:**
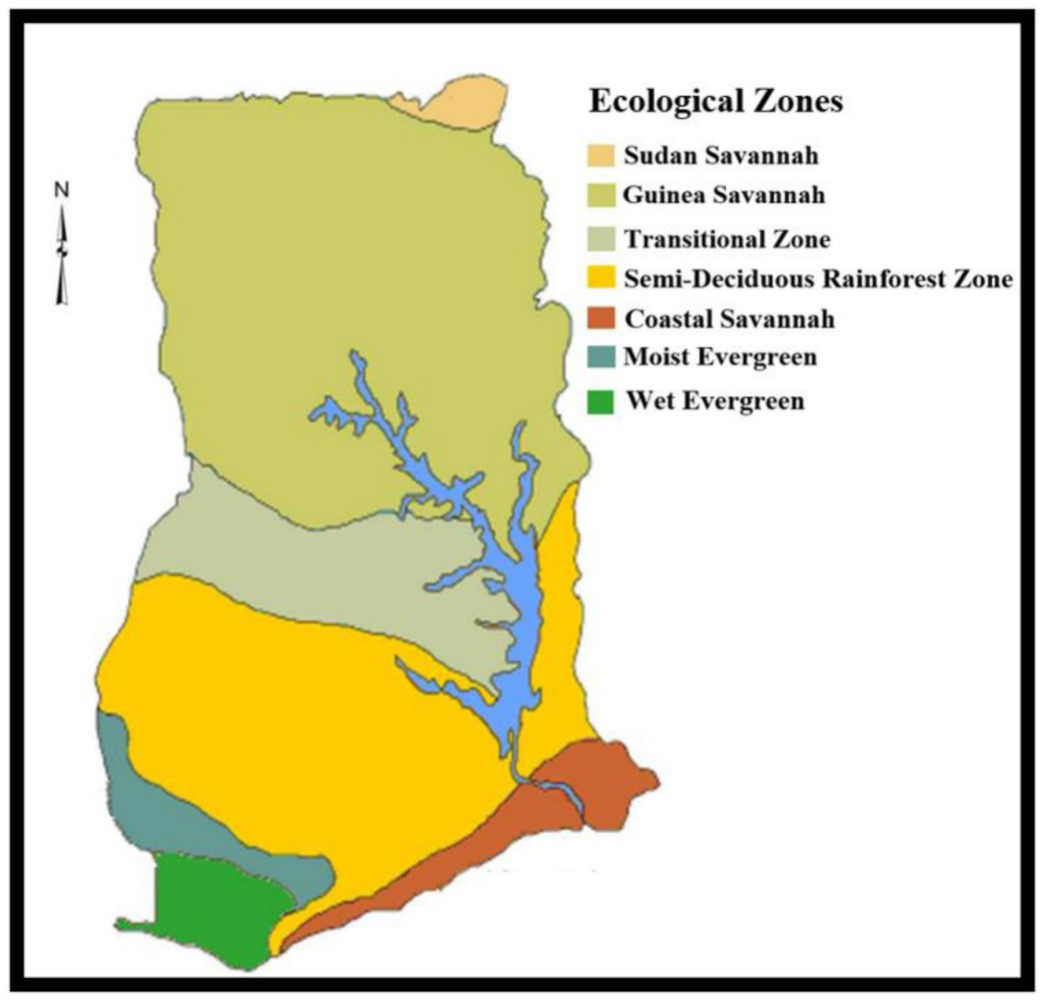
Agroecological zones of Ghana.

The field work was carried out in three agro-ecological zones of Ghana (the Guinea Savannah, Semi-Deciduous Rainforest, and Coastal Savannah) (Fig 1). The Guinea Savannah zone lies between longitude 100 1’ W and latitudes 100 3’ and 110 10’ N. The climate of the location is normally dry with a unimodal rainfall pattern that starts from April to October. The average annual rainfall ranges from 800 to 1200 millimeters. The dry season runs from November to March/April, with the hottest temperatures occurring near the conclusion of the season. The average temperature is 32°C and the average relative humidity (RH) is 33%.

The Semi-Deciduous Rainforest is found in the country’s middle belt and is located between longitude 2.250 W and 0.150 W and latitude7.460 N and 5.500 N. The average yearly rainfall is between 1200 and 1600 millimeters. The zone experiences two rainy seasons: the major season runs from March to July, and the minor season runs from September to November. RH varies from 97% and 20% through to the morning and late afternoon, respectively, with an average temperature of about 27°C. The climate of the Coastal Savannah is a tropical type. Temperatures are regularly high, with very little difference during the year. The yearly average temperature is 26.80°C, with monthly temperatures ranging from 24°C in August to 29°C in March. RH is generally high in the area and varies from 65% in the midday to 95% at night. The bimodal rainfall pattern in the zone gives rise to two rainy growing seasons with an average yearly rainfall of 800mm. The major rainy season runs from May to mid-July, and the minor rainy season runs from mid-August to mid-October.

### Phenotypic traits

Phenotypic traits measured on all (104) birds at 36 weeks of age were: body weight (BW), body length (BL), body width (BWd) and shank length (SL). BW was determined as the weight of the bird using electronic weighing scale. BL was measured from the back of the neck through to the tip of the tail feathers. BWd was measured across the shoulders or wings of the birds. SL was determined from the hock to the spur. These traits were determined because they are key quantitative traits recommended by FAO for phenotypic characterization of indigenous birds (FAO, 2012).

### Collection of blood sample and genomic DNA extraction

Whole blood was sampled from all chickens. Total genomic DNA was extracted from chicken whole blood using the QIAGEN^®^ Kit (QIAGEN. Valencia, CA, USA) at the Molecular Genetics Laboratory of the Department of Animal Science, University of Ghana, Legon. The DNA concentration in the collected samples was determined using a Life Technologies Qubit 3.0 fluorometer. The Qubit was used for evaluating DNA quality prior to next-generation sequencing (dsDNA). According to the manufacturer’s procedures, a Qubit dsDNA BR (wide range, 2 to 1000 ng) Assay Kit and Qubit dsDNA HS (high sensitivity, 0.2 to 100 Nanograms) Assay Kit were used with a Qubit 3.0 fluorometer; a sample volume of 1 μL was added to 199 μL of a Qubit working solution.

### Genotyping chicken accessions using SNP markers

DNA samples from 90 chickens were sent to the Biosciences Eastern and Central Africa (BecA)-ILRI Hub, Nairobi, Kenya (https://www.ilri.org/research/programs/beca-ilri-hub) for genotyping by sequencing (GBS) using DArTseq^™^ technology on Illumina HiSeq2500. Sequencing data was obtained from 62 indigenous chicken ecotypes (S1 Table) and 26 commercial crossbred individuals (S2 Table). DarTsoft14, an automated genotypic data analysis application, and DArTdb, a laboratory management system, were used to examine the sequencing data (Diversity Arrays Technology P/L, Canberra, Australia). The sequences were aligned to the Chicken Reference Sequence, Ggal 5 to identify chromosomes and positions. SilicoDArT SNP identification was performed with MSQ 0.6.6. Only readings that aligned to a single unique site of the genome were considered for SNP finding. SNP markers were scored ’1’ for presence, and ’0’ for absence, and ’-’ for failure to score. To calculate the marker data’s reproducibility, two technical replicates of the DNA samples were genotyped.

## Statistical analysis

### Analysis of phenotypic data

Two-way ANOVA was conducted on the phenotypic data using the 12th edition Genstat statistical software [30], significance was established at p<0.05, and differences between treatment means were differentiated using the Tukey’s Studentized Range Test.

### Analysis of sequencing data

#### Quality analysis of SNP marker data

A total 85,396 SNP markers were generated. The quality control of the markers consisted of excluding those with missing data scaffolds with no specific chromosomal assignment, minor allele frequency of less than 5%, sex chromosomes, and chromosomes with no positions. The “R” software (Hierfstat) was used to test the markers for reproducibility (%), call rate (%), polymorphism information content (PIC), heterozygosity and one ratio [31]. The proportion of technical replicate assay pairs was used to score reproducibility. The call rate was calculated using the percentage of samples with a score of ’0’ or ’1’ to determine the outcome of reading the marker sequence across the samples. Polymorphic information content is the degree of diversity of the marker in the population and shows the usefulness of the marker for linkage analysis. One ratio constitutes the proportion of the samples for which genotype scores equaled ’1’. The PIC refers to the marker’s degree of diversity in the population, and it demonstrates the marker’s usefulness in linkage analysis. The fraction of samples with genotype scores of ’1’ is represented by one ratio.

#### Population structure and genetic diversity

Analysis of population structure was conducted in the R software (Hierfstat) [31]. Nei’s distances were computed to estimate genetic differences in proportion to time. Nei’s genetic distances were used to calculate Analysis of Molecular Variance (AMOVA). The AMOVA, as implemented by the "poppr.amova" function, was used to determine variance within and between the chicken populations. The “stampp F_ST_” function was used to calculate genetic differentiation (F_ST_) across all populations’ pairs. Using principal component analysis (PCA) with the "find. Clusters" function in R, sampling sites investigated the genetic structure, population differentiation, and AEZ depending on the available allelic frequency. Clustering was done using the "aboot" function from the “poppr” package. Discriminant Analysis of Principal Components (DAPC) analysis was employed to identify and describe the number of clusters of genetically related individuals. DAPC plot emphasizes between-group variation as well as within-group variation. The contributions of alleles to the DAPC groupings aid in identifying regions of the genome that drive genetic divergence between populations [32]. A dendrogram was generated for the population differences in the AEZs and individuals were categorized into a predetermined number of population groups (K).

The software program STRUCTURE was used to apply a Bayesian methodology based on the genotypes of the individuals collected [33]. Using a probabilistic approach, individuals in the dataset were assigned to K (unknown) populations. Individuals are allocated membership to all of the distinct clusters (number of clusters = K), where the sum of the probabilities belong to a population equals one (1), and K varies between runs of the program. To ensure a random starting point for the algorithm, STRUCTURE was run with 106 iterations and a burn-in period of 10,000 iterations. To ensure that the findings were consistent, the runs were performed 20 times for 2 greater than (>) K less than (<) 10. A FastStructure, an alternative to Structure, was created specifically for huge SNP datasets [34], like the admixture, ancestry model, which assumes neither polyploid nor dominant data and allows individual birds to have mixed ancestry. This is modeled by assuming that a certain individual has acquired some portion of its genomic DNA from ancestors in population K. This allowed for a graphical representation of the data to be created in order to distinguish between different groups.

## Results

### Phenotypic characterization

The phenotypic characteristics of the chickens studied are presented in Table 1. The chickens from the Semi-deciduous rainforest were heavier and broader (p<0.05) when compared with their counterparts from the Coastal Guinea Savannah. Body length and shank length were not significant (P>0.05) among the chickens from the three AEZs. There was sexual dimorphism among the commercial crossbreds located at KNUST. The males were had higher body weight, body width, body length and shank length (p<0.05) when compared with the females.

**Table 1:** Performance of indigenous chickens from the Coastal Savannah (CS), Semi-Deciduous Rainforest (SDR), Guinea Savannah (GS) and commercial crossbred (CC) chickens located at Kwame Nkrumah University of Science and Technology (KNUST) in Ghana at 36 weeks of age.

Location	Body weight (g)	Body length (cm)	Body width (cm)	Shank length (cm)
CS	846^b^	35.08	7.63^a^^b^	8.00
SDR	1,425^a^	34.38	7.85^a^	8.21
GS	962^b^	35.21	7.21^b^	7.88
SEM	113	0.94	0.23	0.27
Pr>F	0.001	0.645	0.026	0.495
CC				
Male	2,437^a^	53.70^a^	14.81^a^	11.81^a^
Female	1,862^b^	39.02^b^	11.14^b^	9.05^b^
SEM	0.022	0.099	0.278	0.092
Pr>F	<0.001	0<.001	<0.001	<0.001

^a-b^Means in the same column with different superscript are significantly different at P<0.05

### Discovery and quality analysis of SNP markers

**Fig 2:**
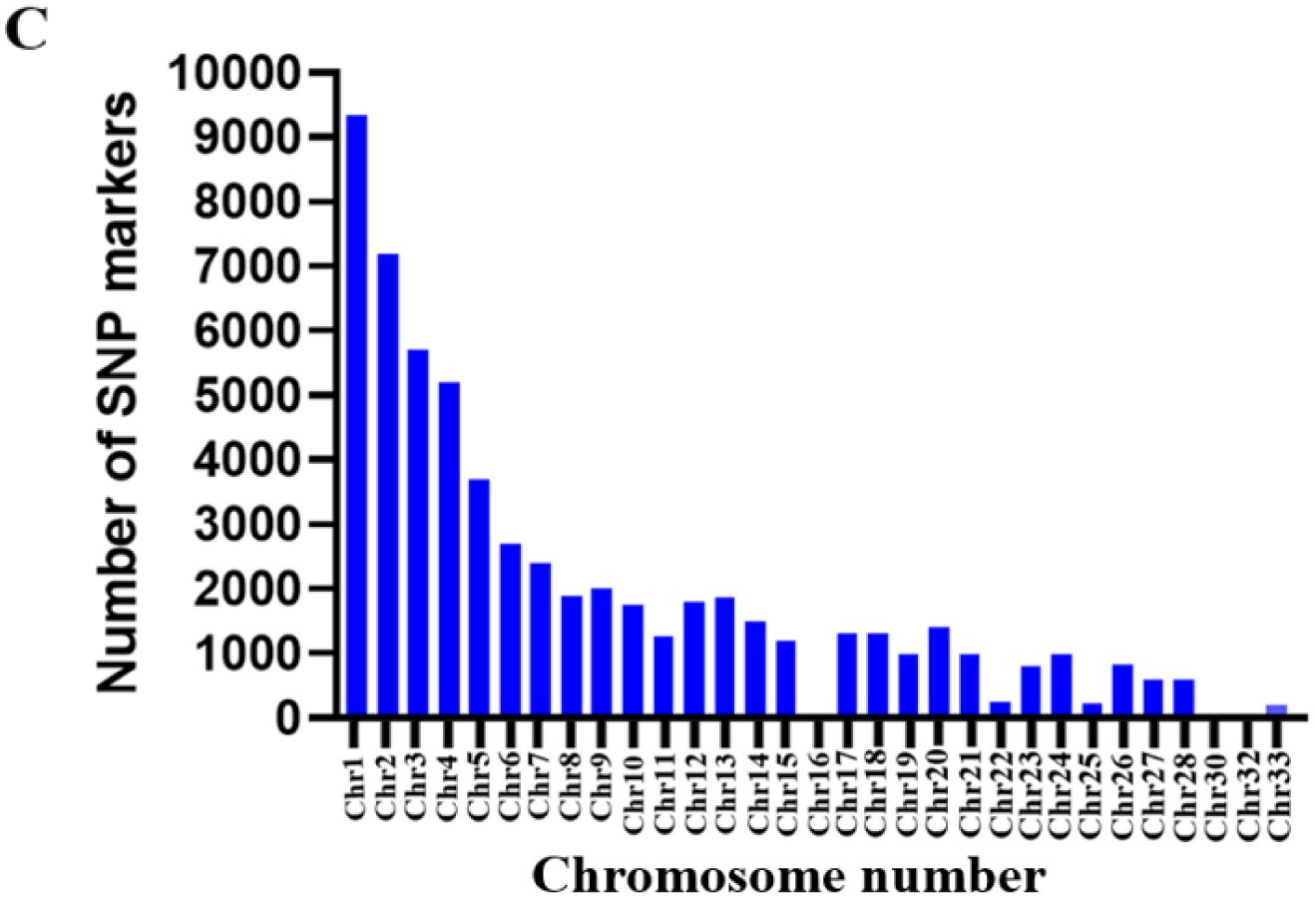
Qualities and characterization of SNP markers in chicken genotype. Call rate of SNP markers tested in chicken genotypes (S1A Fig). Frequency distribution of PIC values for SNP markers (S1B Fig). **Distribution of SNP markers on the chromosome of chicken** (C).

A total of 85,396 SNP markers were generated. After removing markers with >30% missing data scaffolds with no specific chromosomal assignment, minor allele frequency of less than 5%, sex chromosomes, and chromosomes with no positions, 58,353 SNP markers remained for further analysis. The quality of SNP markers was assessed using call rate and PIC. Approximately 70% of SNP markers were more than 95% reproducible and remained within 90% to 95% range. The call rate of 58,353 SNP markers varied from 30% to 100%, with 70% showing ≥ 80% call rate and the remaining markers showing < 80% call rate (S1A Fig). The lowest call rates of the remaining <30% markers were not considered during the analysis. The PIC values for the SNP markers ranged from 0.00 to 0.50. Around 21,285 SNP markers (35%) were in the lowest PIC value range (0 to 0.15), while 13,511 (21%) were in the highest PIC value range (0.45 to 0.50) (S1B Fig). The majority of SNP markers, 48,644 (83%) were found on chromosome 1 to 15, with the remaining markers, 9,709 (17%) found on chromosome 16 to 33 (Fig 2). Moreover, few SNP markers were found on chromosome 16, 31, and 32, with no SNP markers on chromosomes 29 and 30. These findings indicate that SNP markers are of high quality and may have a significant impact on the chickens’ genome.

Analysis of heterozygosity revealed low diversity within the populations studied, with *He* values per population ranging from 0.198 to 0.22 while *Ho* values per population ranged from 0.125 to 0.138. The highest He value was observed in the CC and the lowest in the GS (Table 2). The F_IS,_ which measures the inbreeding of an individual within a sub-population was moderate ranging from 0.352 to 0.442 across all the populations (Table 2).

**Table 2:** Heterozygosity studies among chickens from Coastal Savannah (CS), commercial crossbreds (CC), Semi-deciduous Rainforest (SDR) and Guinea Savannah (GS).

Population	Ho	He	F_IS_
CS	0.125	0.217	0.442
SDR	0.138	0.212	0.367
CC	0.131	0.220	0.419
GS	0.132	0.198	0.352

Ho, Observed heterozygosity; He, Expected heterozygosity; F_IS_, Inbreeding coefficient

### Genetic diversity among population structure

The pairwise F_ST_ statistic among population is used to measure the genetic distance among populations [35]. F_ST_ of the AEZs/ecotypes based on SNPs was performed to obtain the total genetic variance contained in a subpopulation relative to the total genetic variance (Table 3). Between commercial crossbred lines and the indigenous ecotypes, pairwise F_ST_ values were estimated as 0.105 between Coastal Savannah lines, 0.096 between Semi-Deciduous Rainforest lines, and 0.133 between Guinea Savannah lines. Besides, the highest F_ST_ value obtained from pairwise comparison of the three local ecotypes was 0.031 between Guinea Savannah and Semi-Deciduous Rainforest lines; and the lowest F_ST_ value obtained was 0.015 between Semi-Deciduous Rainforest lines and Coastal Savannah lines. We found that the highest level of genetic differentiation was between commercial crossbred chicken breeds and Guinea Savannah zone populations (F_ST_ = 0.133). These findings indicate that there is high genetic structure between commercial crossbreds and indigenous lines from the three AEZs.

**Table 3:** Pairwise F_ST_ among chickens from Coastal Savannah (CS), Commercial crossbreds (CC), Semi-deciduous Rainforest (SDR) and Guinea Savannah (GS) based on SNP data.

	CS	CC	SDR
CS			
CC	0.105*		
SDR	0.015	0.096*	
GS	0.026	0.133*	0.031

*Statistical significance at (P<0.05)

An agglomerative hierarchical cluster analysis was performed on SNP markers using Nei’s genetic distances to study the genetic relationships between the chicken genotypes. The Nei’s genetic distance measures the extent of genetic variation that exists between species. Herein, chickens from the various AEZs, including the commercial crossbreds, were considered. The Nei’s genetic distance values were lower than 0.068 in all populations (Table 4). on the SNP calls. The commercial crossbreds and the Coastal Savannah chickens had a proportionate differentiation of 0.057, and that of the commercial crossbreds and Semi-Deciduous Rainforest was 0.053, and that between the commercial crossbreds and Guinea Savannah was 0.068. In a similar manner, the genetic distance between the Coastal Savannah and Semi-Deciduous Rainforest lines was 0.019, Guinea Savannah and Semi-Deciduous Rainforest was 0.025, as well as 0.021 between the Coastal Savannah and Guinea Savannah. These findings showed that there is more genetic overlap between the commercial crossbreds and Guinea Savannah chickens, followed by the commercial crossbreds and the Coastal Savannah chickens.

**Table 4:** Nei’s genetic distance among chickens from Coastal Savannah (CS), Commercial crossbreds (CC), Semi-deciduous Rainforest (SDR) and Guinea Savannah (GS) based on SNP data.

	CS	CC	SDR
CS			
CC	0.057*		
SDR	0.019	0.053*	
GS	0.021	0.068*	0.025

*Statistical significance at (P<0.05)

### Dendrogram analysis

**Fig 3:**
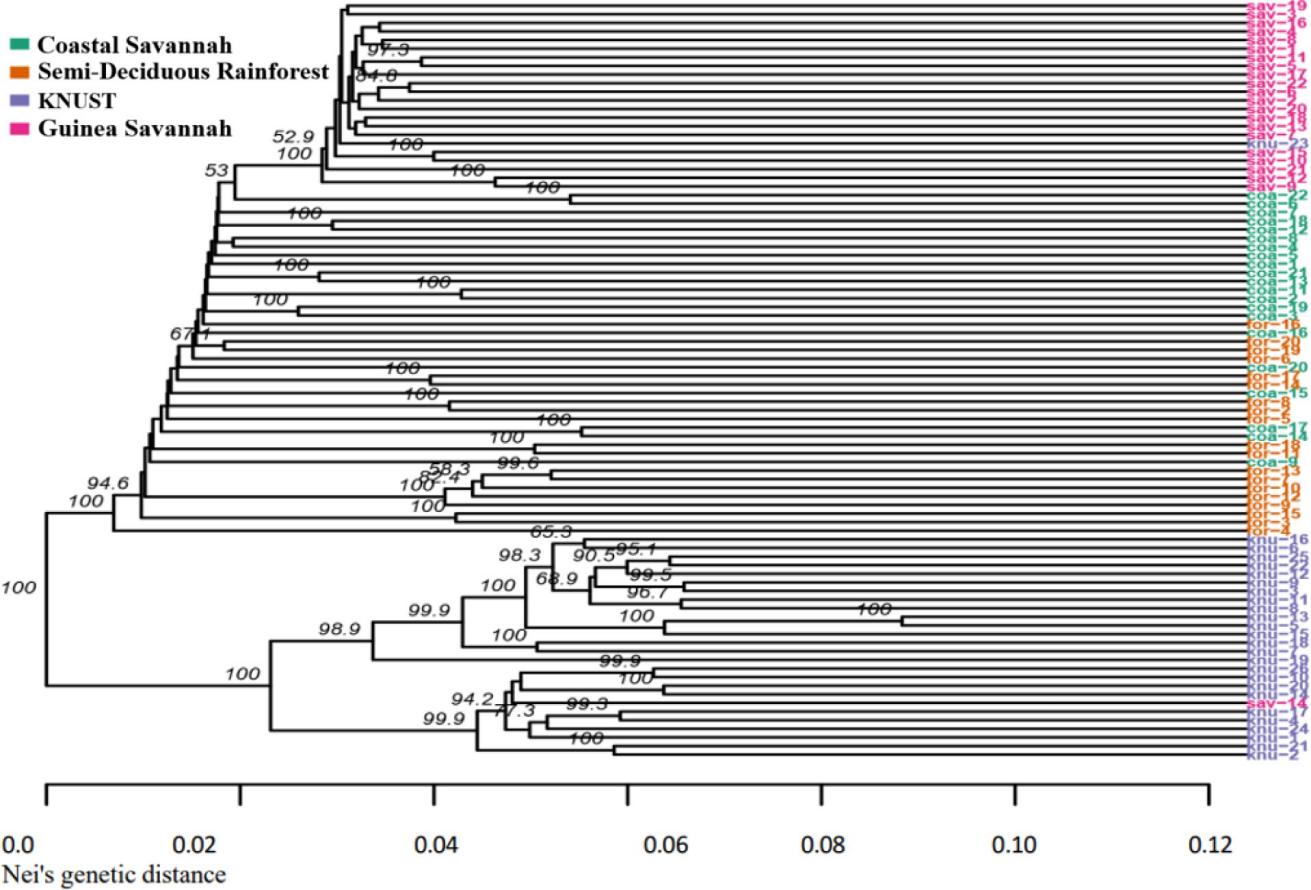
Dendrogram showing relatedness among chicken genotypes based on SNP markers.

The dendrogram generated was based on the Nei’s genetic distance [36] among individuals within the chicken (Fig 3). Besides, the clustering based on the SNP markers produced two main clusters of related populations or similar origin. Cluster 1 consisted of all the commercial crossbreds. The clustering within the commercial crossbreds had various degrees of similarity, ranging from a high similarity (100%) to a lower similarity index (68.9%). Two individual birds from the commercial crossbreds identified as “KNU 13” and “KNU 5” are the least diverged individuals among this cluster, with Nei’s genetic distance of 0.09 and a high similarity index of 100%.

The indigenous ecotypes were considered as one cluster however, the Guinea Savannah population formed a sub-group within the cluster. From the current data, the SNP markers data showed a shorter Nei’s genetic distance ranging from 0.00 to 0.12. While the Semi-Deciduous Rainforest and Coastal Savannah populations tended to cluster together, it appeared that Semi-Deciduous Rainforest, Coastal Savannah, and Guinea Savannah populations shared most SNP alleles and were less diverged. However, they also shared a degree of similarity ranging from high similarity (100%) to low similarity index (52.9%). However, the commercial crossbreds had a wide range of diversity as compared to the other populations.

### Detection of number of clusters and principal component analysis

**Fig 4:**
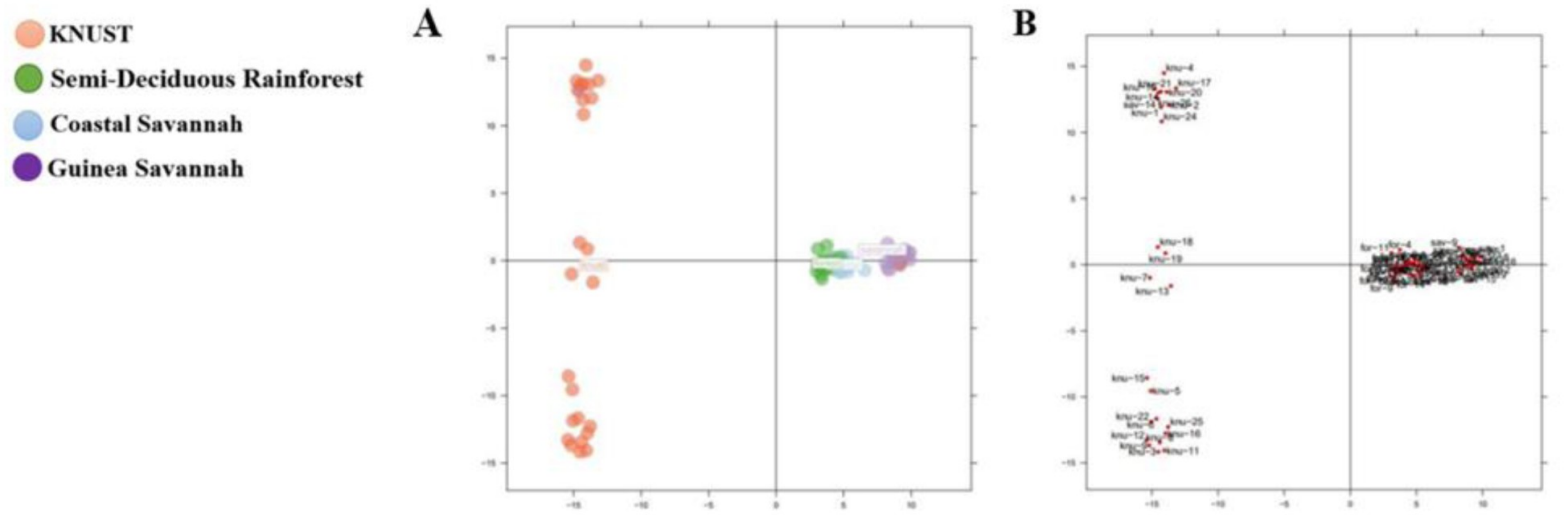
Principal component analysis of the chicken genotype based on SNP markers. The optimum K-value was 2.

To confirm the separation based on commercial crossbred chickens (KNUST) and chickens from the three ecological zones, the data was subjected to PCA plot to show the presence of potential clusters of populations, with principal components jointly explaining the total genetic variance in the chicken populations using the SNP data. Score scatter plots showed the differentiation and similarities among the chickens (Fig 4A and 4B). The first principal component highlighted the genetic variation between the chicken populations. We separated the cluster comprising chicken populations belonging to the Semi-Deciduous Rainforest, Guinea Savannah, and Coastal Savannah zones from the commercial crossbred chickens, which had extended to two main different clusters. The commercial crossbred population was more dispersed and appeared not to share much similarity with the chicken ecotypes for the three AEZs. This analysis shows that chickens from KNUST have a diverse genetic composition when compared to indigenous chickens from the ecotypes.

### Analysis of molecular variance

Analysis of molecular variance (AMOVA) was conducted to partition the molecular diversity among and within populations for SNP marker data. As presented in Table 5, the AMOVA revealed that the highest level of variation (82%) was within the samples’ variance. However, types within the population had the lowest variation (1.062%). Also, the variation between the chicken populations was 6.9%.

**Table 5:** Analysis of molecular variance based on SNP marker data.

Source of variation	DF	SS	MS	Sigma	Variance (%)
Between chicken population	4	22,777	5,694	112	6.92
Between type with chicken population	9	16,723	1,858	17	1.06
Between samples with type	74	122,409	1,564	159	9.76
Within samples	88	117,655	1,337	1,337	82.26
Total	175	279,564	10,453	1,625	100.00

**Fig 5:**
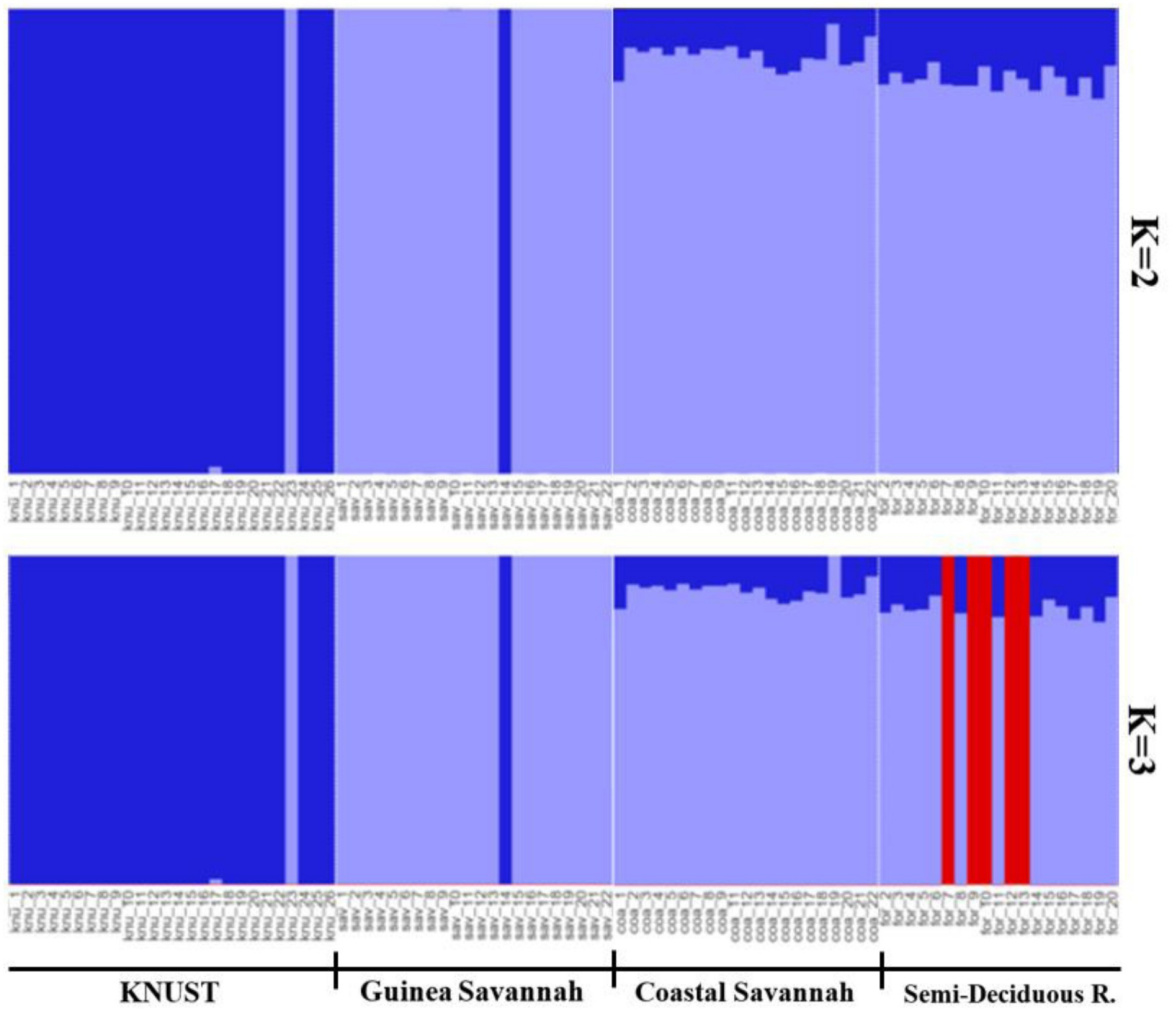
A structure bar plot showing the assignment of indigenous chicken and commercial crossbred (KNUST) populations. Each vertical bar represents one population and different colors within each bar indicate admixture. (K = 2), K means the number of groups.

Results from the population structure analysis are shown in Fig 5. A plot against number of suitable clusters for chicken populations which was estimated from the SNP markers for CS, GS, SDR and KNUST showed that, the lowest Bayesian Information Criterion (BIC) value was obtained at K = 3. Therefore, two discrimination function were detected, which showed that the chickens studied belonged to three (3) potential groups. This was employed through Discriminant Analysis of Principal Component (DAPC).

The analysis indicates that the best grouping of the population was two (K = 2). A total of 26 individuals, mainly from the commercial crossbreds (KNUST) were entirely grouped in population 1 (deep blue), while 22 chickens mainly from Guinea Savannah were grouped in population 2 (light blue). Forty chickens mostly from Coastal Savannah and Semi-Deciduous Rainforest ecotypes were in the admixture group.

The population structure and the number of real chicken groups within the population studied have been shown in Fig 5.

## Discussion

Current studies indicate that the use of SNP markers has significant role to play in determining genetic diversity and population structure in animal species, such as in amphibians [37], grasshoppers [38], fishes [39] and lobsters [20] and chickens [26, 40]. For example, SNP data is known to provide more reliable inferences of patterns of genetic structure and diversity than a typical microsatellite dataset in Liberian amphibians [37]. In addition, Dementieva et al. used SNP markers to analyzed genetic diversity of different chicken breeds [40]. Therefore, studying the genetic diversity and population structure of chickens from three AEZs and KNUST using SNP markers, may contribute to the understanding the diversity and structure of chickens reared in different AEZs in Ghana. In the current study, we found that there is high genetic diversity and population structure between the commercial crossbreds and indigenous chickens across the AEZs.

The current study aimed at investigating some of the phenotypic differences among chickens located in three AEZs (Coastal Savannah, Semi-deciduous Rainforest and Guinea Savannah) and commercial crossbreds located at KNUST. There are different AEZs in Ghana, and these can directly and indirectly affect the survival [41], productivity [42, 43], and adaptation [44] of animals. Free-roaming birds may perform better or poorly based on the resources available in the AEZ where they live. [45]. The climate of the Guinea Savannah AEZ is normally dry with a unimodal rainfall pattern. The average annual rainfall ranges from 800 to 1,200 millimeters. The average temperature is 32°C and the average relative humidity (RH) is 33%. The people here engage more in ruminant farming than crops. Feed resources for free-roaming birds is limited within this AEZ. The Coastal Savannah AEZ has a bimodal rainfall pattern with an average of 800mm. The yearly average temperature is 26.80°C. Relative humidity is generally high in the area and varies from 65% in the midday to 95% at night. These are the coastal areas of Ghana, and the people are mainly fisher men and fish mongers, few engage in crop farming in addition to fishing. Availability of feed resources for free-roaming birds is moderate. The Semi-Deciduous Rainforest (SDR) is found in the country’s middle belt. The yearly bimodal rainfall is between 1200 and 1600 millimeters. Relative humidity varies from 97% and 20% with an average temperature of about 27°C. This is a forest zone with green vegetation available throughout the year with the availability of different species of worms and insects and their larvae. The people located in the SDR are mainly crop farmers and therefore crop residues and other crop-based by-products are readily available which makes this AEZ resource-rich for free-roaming birds. Among the local ecotypes, chickens from the Semi-deciduous rainforest were the heaviest compared to their counterparts in the coastal Savannah and the Guinea Savannah. Most local chicken ecotypes are reared in the backyard and rely primarily on scavenging for their nutrition. Household scraps and agro-byproducts are their main source of nutrition. The yearly average rainfall of Semi-deciduous Rainforest (1,200–1,600 mm) is adequate for the cultivation of large-scale plantation crops such as cocoa, oil palm and lemon, as well as, annual crops (maize, cassava and plantain) [46]. By products from such crops, insects and worms form a bulk of their diet. The abundant agro-byproducts in the Semi-deciduous rainforest contribute to the better growth of chicken ecotypes in that AEZ compared to their counterparts in the Savannahs. These account for the significant increase in body weight and width of chickens from the Semi-deciduous rainforest compared to the other AEZ. The Semi-deciduous rainforest may be the most suitable environment to raise indigenous chickens whose sustenance depend primarily on scavenging.

Currently, data on phenotypes, genetic diversity and population structure among chickens in different AEZs in Ghana is scanty. Through the application of DArTseq genotyping, a total of 58,353 reads of SNP markers were discovered and used for genetic diversity characterization in indigenous and commercial chickens. SNPs form the basis for large DArTseq genotyping platforms [47] and can also contribute to the forthcoming version of the chicken genome. The SNP markers used in this study showed an average genotype call rate of 90%, showing that the quality of the SNPs was high. The average call rate of 90% in this study presented by SNP markers was lower than the 97% and 98.7% observed in the study of 55K SNP genotyping array in different breeds in China [48].

The PIC value reveals information about the diversity. PIC values can be categorized into three: high (0.40 to 0.5), moderate (0.10 to 0.25), and low (0 to 0.10) [49, 50]. In the current study, 35% of SNP markers were in the lowest PIC value range (0 to 0.15), while 21% were in the highest PIC value range (0.45 to 0.50), with an average value of 0.26. Thus, about 67% of the SNP markers discovered showed moderate to high polymorphism and could be used for other genome wide studies. The study by Liu et al. had 76.7% to 88% of SNPs being polymorphic compared to the 100% of SNPs showing polymorphism in current study [48]. A similar situation was observed when DArT seq SNP markers were used for genotyping-by-sequencing in horses (96.7%), cattle (95.4%) and sheep (97%) [51]. The higher polymorphisms observed in our study is attributed to the quality screening process applied in our study. The main criteria in the study in the China was to discard the markers with Minor Allele Frequency (MAF) ≥ 0.05 while in horses, cattle and sheep markers with MAF> 0.01 were discarded; these markers were also filtered based on default parameters set in the FastQC software, missing genotype (n > 20% of individuals) and deviation from Hardy-Weinberg equilibrium with p < 0.001. However, DArTseq SNP marker in our study showed a higher polymorphism due to high quality filtering measures employed such as using the filter option of MS Excel for filtering, and also the next-generation sequencing technology used allows for high confidence in marker calling and estimates of minor allele frequency (MAF) by counting the occurrence of the two variants.

The DArTseq markers (SNPs) used in this study had an average PIC value of 0.26, which was lower than what was observed in Korean and Iranian native chicken using microsatellite markers [52, 53]. This difference in genome coverage and PIC of markers was observed in other studies that used DArTseq markers. A PIC between 0.50 was presented by 20% of DArTseq SNP markers in macadamia [54]. The study in cassava revealed the average of 0.28 for DArTseq SNP markers [55] and respective PIC average of 0.18 in Napier grass [56]. This was confirmed by their mapping on chicken chromosomes. The polymorphic markers were mapped differently on chicken chromosomes, where DArTseq SNP markers were mapped on 29 chromosomes. Moreover, when compared to some studies in animal species where SNP markers were used, the average PIC value was similar, and in some cases higher than what was observed in these animals. For example, in Berkshire pigs, it was 0.26 ± 0.11 [57], while in a study of two exotic layer chicken strains it was between 0.1 and 0.2 [58]. This indicates that the SNP markers from the study were significantly informative for genetic diversity and genome wide association studies in chickens. Considering the heterozygosity, low levels of H_O_ (ranging from 0.125–0.138) in the analyzed populations in this study point to high heterozygote deficiency. Our study showed that, the expected heterozygosity value for the CC (0.220) was higher than those obtained by Dalirsefat et al. on White Leghorn (0.19) using SNP marker [59]. This indicates that the CC are genetically diverse when compared to the White Leghorn as well as the indigenous populations from the three agro-ecological zones studied in our research.

The low F_ST_ values among the indigenous ecotypes indicate low genetic differentiation among these populations, which means they might have a common origin. However, there were high F_ST_ values among the crossbreds at KNUST and the indigenous chicken ecotypes. Considering the Nei genetic index, we observed a higher genetic diversity between the crossbreds located at KNUST and the indigenous groups, which could be attributed to the differences between the exotic parental types of the crossbreds and the indigenous parental type. The crossbreds located at KNUST and the Coastal Savannah chickens had a proportionate differentiation of 0.057 suggesting that these two chicken populations may have some overlap. their DNA segments. This suggest that the crossbred may share ancestry with some of the chickens in the Coastal Savannah. The genetic differentiation between the Coastal and Guinea Savannah and that of Coastal and Semi-Deciduous Rainforest had the lowest estimated distance. This implies that there is less genetic diversity between the populations with shorter genetic distances, which in turn mirrors the population history with a likely common origin. The dendrogram between commercial crossbred chickens (KNUST) and indigenous chickens from different AEZs were based on significant SNP markers (Fig 3). The dendrogram based on genetic distances shows that there is a clear difference between KNUST (group 1) and indigenous chickens from the AEZs (group 2). This clearly corroborates the F_ST_ results that shows that the commercial crossbreds cluster differently from the indigenous ecotypes. The information generated shows a detailed understanding of the genetic relationship among the chicken clusters. The outliers that appeared in KNUST and GS chickens may have been caused by labeling errors during DNA extraction or sequencing. One sample from KNUST was exchanged with one sample from GS. Some birds of CS origin were found in SDR because CS shares a boundary with SDR and there is frequent movement of birds between the two zones. It is, therefore, possible that some of the birds sampled from SDR were birds originated from CS.

Additionally, we used the PCA method to ascertain differences and similarities among the different groups of chickens [60]. We ascertained that chickens from the three AEZs were located in the same areas of the loading plot and clustered together, indicating good repeatability of the test and a putative common origin. However, the commercial crossbreds located at KNUST were clearly separated from the indigenous chickens in the loading plot. We used AMOVA to examine the genetic variation among and within the different chicken groups [59]. The current study revealed that there was 6.92% genetic variation between the chicken population and that most of the variation came from variation within samples among populations. Similar outcome was observed in four Thai indigenous chickens [61]. The current study showed arrangement in structure output at K = 2 which suggests a high genetic differentiation among the studied chicken populations and once again, the chickens from the Guinea Savannah, Semi-Deciduous Rainforest, and Coastal Savannah AEZs clustered together and are farther away genetically from the commercial crossbred chickens at KNUST.

The close relationship among the indigenous genotypes was not surprising as it is known that most of the indigenous chicken in Ghana share their parentage from the Red Jungle Fowl (*Gallus ferrugineus or bankiva*), which was developed in Asia [62, 63]. From the dendrogram it can be observed also that, among indigenous chicken in Ghana, the Semi-Deciduous Rainforest population appears to be genetically richer than the Guinea Savannah population and some of the Coastal Savannah genotypes can be found within the SDR population. Osei-Amponsah et al. (2010) had a similar finding attributed it to the reason that, the Ghanaian indigenous chickens were introduced into the country during the precolonial period through trade with early European navigators who arrived at the coast of Ghana [28]. The early traders, who were mainly interested in gold and cocoa, made contacts with local people along the coast and the Forest areas of Ghana. As a result, the original chicken population was most likely located in the Forest belt of Ghana and it subsequently spread to other areas of the country through trade and movement of people between Ghana’s Forest and Savannah zones. Many migrant labourers moved from the Northern Savannah to the Forest zone to work on cocoa plantations and gold mines [64], and these may have helped to spread the birds from the SDR zone to CS zone. Thus, despite the fact that they relocated further from their initial area in the Forest to the Savannah causing the dilution of genes due to breeding with other populations, traces of the similarity still remain within these two ecozones and this study is in support of these observations, which were also observed by other researchers based on mitochondrial DNA diversity studies [65–67].

From the above discussion, indigenous chicken ecotypes from the three AEZs, according to the genetic diversity parameters such as F_ST,_ Nei genetic index, dendogram and PCA, have a common origin and are genetically close. This is an indication that the differences recorded in the phenotypic characterization were due to the differences in climate and availability of feed resources for free-roaming chickens in the various AEZs. Therefore, any future plans to select indigenous chickens in Ghana for improved performance in various economic traits should critically consider the different AEZs pertaining in the country as well as the various poultry production systems existing in these AEZs.

## Conclusion

In the current study, we investigated the genetic structure and variability in chickens from three AEZs and commercial crossbreds raised at the Kwame Nkrumah University of Science and Technology (KNUST) using SNP markers. Here, we demonstrated that the chickens from the Semi-Deciduous Rainforest, Coastal Savannah and Guinea Savannah may have a common genetic origin and can be considered as one genetic population. This confirms the fact that indigenous chickens in Ghana share a common parentage from the Red Jungle Fowl, and over the years movement of birds across the ecological zones through trade and resettlement has led to random mating between birds from the various AEZs and this explains the genetic closeness among birds from the three AEZs. These local ecotypes are different in structure and are genetically distant from the commercial crossbred chickens reared in KNUST, which is a product of crossbreeding between Ghanaian local chickens and exotic commercial birds. They were backcrossed with the commercial birds for six more generations and have been raised on-station under intensive system for over ten years. As a result of the continues crossing with exotic commercials, physiological adaptation to the intensive rearing system and epigenetics, these crossbreds are now genetically distant from the Ghanaian indigenous chickens. Indigenous birds from the three AEZs are genetically close but due to the abundant agro-byproduct resources in the Semi-Deciduous Rainforest, the chickens in this AEZ had better growth than the chickens reared in the Savannahs. The AMOVA analysis also showed that there is ample room within the sample genetic variability suggesting that, these indigenous populations may not be under immediate threat of inbreeding and can play a significant role in future genetic improvement programs. This study has shown that although there is minimal genetic introgression of exotic poultry into the indigenous chickens in the three AEZs, to develop a sustainable chicken breeding scheme in Ghana, it will be important to preserve the native genetic resources.

## Supporting information

S1 Fig A: Call rate of SNP markers tested in chicken genotypes, B: Frequency distribution of PIC values for SNP markers. (ZIP)

S1 Table List of indigenous domestic chickens used in the genetic diversity. (DOCX)

S2 Table List of commercial chickens used for the genetic diversity study. (DOCX)

S1 Data (RAR)
