# Supplementary figures and images for: Phenotypic characterization and analysis of genetic diversity between commercial crossbred and indigenous chickens from three different agro-ecological zones using DArT-Seq technology
Source: PLoS One. 2024 May 2;19(5):e0297643. doi: 10.1371/journal.pone.0297643 (PMC11065228; doi:10.1371/journal.pone.0297643)

S 1 Figure B: Frequency distribution of PIC values for SNP markers


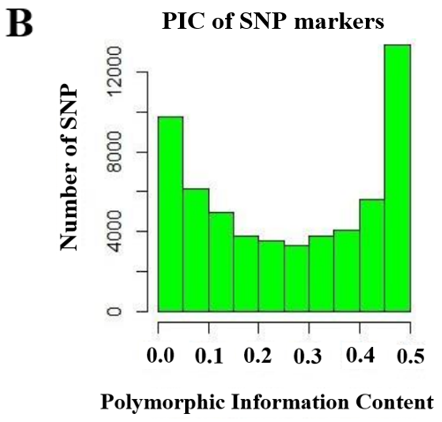

Supplement: S1 Fig — (ZIP) [file pone.0297643.s001.zip › S 1 Figure B.docx]

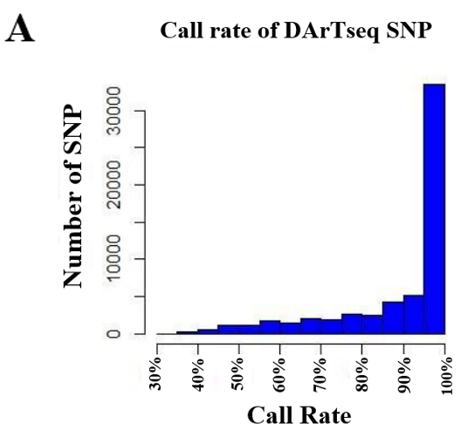
S 1 Figure A: Call rate of SNP markers tested in chicken genotypes

Supplement: S1 Fig — (ZIP) [file pone.0297643.s001.zip › S 1 Figure A.docx]
