# Supplementary material for: Phenotypic characterization and analysis of genetic diversity between commercial crossbred and indigenous chickens from three different agro-ecological zones using DArT-Seq technology
Source: PLoS One. 2024 May 2;19(5):e0297643. doi: 10.1371/journal.pone.0297643 (PMC11065228; doi:10.1371/journal.pone.0297643)
Supplement: S1 Table — (DOCX) [file pone.0297643.s002.docx]

S1 Table: List of indigenous domestic chickens used in the genetic diversity

| Dart.Id | ID | Ecozone | Location | Sex | Color |
| --- | --- | --- | --- | --- | --- |
|  |  |  |  |  |  |
| 3 | coa_1 | Coastal | akotokyir | male | Mottled |
| 94 | coa_2 | Coastal | akotokyir | male | Mottled |
| 104 | coa_3 | Coastal | akotokyir | male | Mottled |
| 1 | coa_4 | Coastal | akotokyir | male | Mottled |
| 58 | coa_5 | Coastal | akotokyir | male | Mottled |
| 41 | coa_6 | Coastal | akotokyir | male | Mottled |
| 56 | coa_7 | Coastal | akotokyir | male | Mottled |
| 32 | coa_8 | Coastal | akotokyir | male | Mottled |
| 97 | coa_9 | Coastal | akotokyir | female | Mottled |
| 7 | coa_11 | Coastal | akotokyir | female | Mottled |
| 9 | coa_12 | Coastal | akotokyir | female | mottled |
| 109 | coa_13 | Coastal | akotokyir | female | mottled |
| 108 | coa_14 | Coastal | akotokyir | female | mottled |
| 17 | coa_15 | Coastal | akotokyir | female | mottled |
| 80 | coa_16 | Coastal | akotokyir | female | mottled |
| 6 | coa_17 | Coastal | akotokyir | female | mottled |
| 75 | coa_18 | Coastal | akotokyir | female | mottled |
| 84 | coa_19 | Coastal | akotokyir | female | mottled |
| 21 | coa_20 | Coastal | akotokyir | male | mottled |
| 59 | coa_21 | Coastal | akotokyir | female | mottled |
| 48 | coa_22 | Coastal | akotokyir | male | mottled |
| 93 | for_2 | Forest | juaso | female | mottled |
| 33 | for_3 | Forest | juaso | female | mottled |
| 112 | for_4 | Forest | juaso | male | mottled |
| 16 | for_5 | Forest | juaso | female | mottled |
| 111 | for_6 | Forest | juaso | female | mottled |
| 39 | for_7 | Forest | juaso | female | mottled |
| 60 | for_8 | Forest | juaso | female | mottled |
| 35 | for_9 | Forest | juaso | female | mottled |
| 67 | for_10 | Forest | juaso | female | mottled |
| 29 | for_11 | Forest | juaso | female | mottled |
| 78 | for_12 | Forest | juaso | female | mottled |
| 10 | for_13 | Forest | juaso | male | mottled |
| 13 | for_14 | Forest | juaso | male | mottled |
| 34 | for_15 | Forest | juaso | male | mottled |
| 2 | for_16 | Forest | juaso | male | mottled |
| 70 | for_17 | Forest | juaso | male | mottled |
| 37 | for_18 | Forest | juaso | male | mottled |
| 66 | for_19 | Forest | juaso | male | mottled |
| 105 | for_20 | Forest | juaso | male | mottled |
| 82 | sav_1 | Savannah | savelugu | female | mottled |
| 15 | sav_2 | Savannah | savelugu | female | mottled |
| 51 | sav_3 | Savannah | savelugu | female | mottled |
| 23 | sav_4 | Savannah | savelugu | female | mottled |
| 63 | sav_5 | Savannah | savelugu | female | mottled |
| 20 | sav_6 | Savannah | savelugu | female | mottled |
| 91 | sav_7 | Savannah | savelugu | female | mottled |
| 107 | sav_8 | Savannah | savelugu | female | mottled |
| 103 | sav_9 | Savannah | savelugu | male | mottled |
| 45 | sav_10 | Savannah | savelugu | male | mottled |
| 4 | sav_11 | Savannah | savelugu | male | mottled |
| 83 | sav_12 | Savannah | savelugu | male | mottled |
| 25 | sav_13 | Savannah | savelugu | male | mottled |
| 53 | sav_14 | Savannah | savelugu | male | mottled |
| 62 | sav_15 | Savannah | savelugu | male | mottled |
| 65 | sav_16 | Savannah | savelugu | male | mottled |
| 49 | sav_17 | Savannah | savelugu | male | mottled |
| 92 | sav_18 | Savannah | savelugu | male | mottled |
| 74 | sav_19 | Savannah | savelugu | male | mottled |
| 43 | sav_20 | Savannah | savelugu | female | mottled |
| 30 | sav_21 | Savannah | savelugu | female | mottled |
| 46 | sav_22 | Savannah | savelugu | female | mottled |
