# Supplementary material for: Phenotypic characterization and analysis of genetic diversity between commercial crossbred and indigenous chickens from three different agro-ecological zones using DArT-Seq technology
Source: PLoS One. 2024 May 2;19(5):e0297643. doi: 10.1371/journal.pone.0297643 (PMC11065228; doi:10.1371/journal.pone.0297643)
Supplement: S2 Table — (DOCX) [file pone.0297643.s003.docx]

S2 Table: List of commercial chickens

| Dart.Id | ID |  | | Location | | | Sex |  | | | Color |  |
| --- | --- | --- | --- | --- | --- | --- | --- | --- | --- | --- | --- | --- |
|  |  | |  | |  |  | | |  |  | | |
| 11 | knu_1 | |  | | knust | female | | |  | white | | |
| 26 | knu_2 | |  | | knust | female | | |  | white | | |
| 77 | knu_3 | |  | | knust | male | | |  | brown | | |
| 57 | knu_4 | |  | | knust | female | | |  | white | | |
| 24 | knu_5 | |  | | knust | male | | |  | brown | | |
| 101 | knu_6 | |  | | knust | male | | |  | brown | | |
| 12 | knu_7 | |  | | knust | male | | |  | white | | |
| 31 | knu_8 | |  | | knust | male | | |  | brown | | |
| 88 | knu_9 | |  | | knust | male | | |  | brown | | |
| 52 | knu_10 | |  | | knust | male | | |  | white | | |
| 69 | knu_11 | |  | | knust | male | | |  | brown | | |
| 36 | knu_12 | |  | | knust | female | | |  | brown | | |
| 28 | knu_13 | |  | | knust | male | | |  | white | | |
| 64 | knu_14 | |  | | knust | male | | |  | white | | |
| 44 | knu_15 | |  | | knust | male | | |  | brown | | |
| 90 | knu_16 | |  | | knust | female | | |  | brown | | |
| 55 | knu_17 | |  | | knust | male | | |  | white | | |
| 38 | knu_18 | |  | | knust | female | | |  | brown | | |
| 99 | knu_19 | |  | | knust | female | | |  | brown | | |
| 98 | knu_20 | |  | | knust | male | | |  | white | | |
| 79 | knu_21 | |  | | knust | female | | |  | white | | |
| 18 | knu_22 | |  | | knust | female | | |  | brown | | |
| 54 | knu_23 | |  | | knust | male | | |  | white | | |
| 85 | knu_24 | |  | | knust | female | | |  | white | | |
| 100 | knu_25 | |  | | knust | female | | |  | brown | | |
| 5 | knu_26 | |  | | knust | female | | |  | white | | |
|  |  | |  | |  |  | | |  |  | | |
